# Calcitonin gene-related peptide predicts therapeutic response to midodrine hydrochloride in children with vasovagal syncope

**DOI:** 10.3389/fnins.2022.1026539

**Published:** 2022-10-04

**Authors:** Lintian Li, Huacai Zhao, Xiuxiu Ma, Fuyong Jiao, Jing Lin

**Affiliations:** ^1^School of Public Health, Xi’an Jiaotong University, Xi’an, China; ^2^Department of Surgical, The Third Affiliated Hospital of Medical College, Xi’an Jiaotong University, Xi’an, China; ^3^Department of Pediatrics, The Third Affiliated Hospital of Medical College, Xi’an Jiaotong University, Xi’an, China; ^4^Key Laboratory for Disease Prevention and Control and Health Promotion of Shaanxi Province, Department of Child and Adolescent Health Science Center, Xi’an Jiaotong University, Xi’an, China

**Keywords:** vasovagal syncope, midodrine hydrochloride, calcitonin gene-related peptide, childhood, predictive study

## Abstract

The vasoconstriction agent midodrine hydrochloride is a vital treatment for pediatric patients diagnosed with vasovagal syncope (VVS), although the efficacy is variable. This study was designed to explore the value of calcitonin gene-related peptide (CGRP) in predicting the effect of midodrine hydrochloride treatment upon VVS patients. In total, 55 children diagnosed with VVS were treated with midodrine hydrochloride for 3 months. Therapeutic response was evaluated using a symptom score system. CGRP levels were significantly higher in VVS patients (68.700 ± 6.460) than in control subjects (43.400 ± 5.810; *t* = 18.207, *P* < 0.001) and symptom scores correlated positively with CGRP concentrations (*r* = 0.779, *P* < 0.001). Patients treated with midodrine hydrochloride showed a significant reduction in symptom scores [4 (0, 6.5) vs. 1 (1, 2); *z* = –6.481; *P* < 0.001]. However, the value of plasma CGRP were potently elevated in the positive-response subjects than in the negative-response subjects (70.080 ± 5.040) vs. (61.150 ± 3.090); *t* = 5.817; *P* < 0.001). The area under the ROC curve showed that the value of CGRP for predicting the therapeutic response to midodrine hydrochloride was 0.946 (95% CI: 0.879–0.997, *P* < 0.001). With high sensitivity (97.7%) and specificity (83.3%), CGRP predicted the therapeutic response to midodrine hydrochloride (cut-off value, 62.56 pg/ml). In conclusion, CGRP can be used to predict the effect of midodrine hydrochloride administration in VVS patients.

## Introduction

Featured by a brief loss of postural tone and a temporary clouding of consciousness and induced by a transient hypoperfusion of the cerebral system, vasovagal syncope (VVS) has mostly been identified in children and adolescents (Grubb, 2005a). It is reported that VVS could be regulated by the autonomic nervous system, and multiple factors could trigger it. Also, VVS is identified to be relative to promoted endothelium-dependent diastolic function, aberrant Bezold–Jarisch reflex, hypoperfusion of the cerebral system, and abnormal autonomic nervous system (Mosqueda-Garcia et al., 2000). Considering the crucial effect of vasodilatation in the pathogenetic development of VVS, midodrine hydrochloride was used to increase peripheral blood pressure by inducing constriction of the arterial and venous beds, and has been shown to be effective in the management of VVS (Sheldon et al., 2021). However, the midodrine hydrochloride treatment response rate is only around 70%. Zhang et al. (2012a) reported that flow-mediated vasodilation (FMD) of the brachial artery can be used as an indicator for selecting midodrine hydrochloride as a treatment for VVS in children. Using a cut-off of 8.85%, the therapeutic efficacy of midodrine hydrochloride was predicted by FMD, with a specificity of 80% and a sensitivity of 90% (Zhang et al., 2012a). However, only 24 children were involved in this study, and more in-depth investigations are needed to verify the aforementioned data and identify additional, more sensitive biomarkers for evaluating the response of patients treated with midodrine hydrochloride.

Widely generated in the nervous systems, calcitonin gene-related peptide (CGRP), a multifunctional neuropeptide consisted of 37-amino acids, profoundly participated in several pathophysiological developments of both the peripheral and central nervous system (Amara et al., 1982). It is a potent vasodilator relative to the regulation of angiotasis (Rosenfeld et al., 1983). Neither species nor tissue dependent (Russell et al., 2014), the vasodilatory effects of CGRP mainly act upon small vasculatures in the peripheral system rather than the great arteries (Sekiguchi et al., 1994). Excessive vasodilatation is known to be a vital pathophysiological mechanism underlying the reduced cardiac output in a group of patients with VVS (Liu et al., 2000; Fu and Levine, 2014). It was proved already that the plasma hydrogen sulfide (H_2_S) which was a vasodilator increased a lot in VVS patients (Zhang et al., 2012b). As an active molecule to dilate blood vessels, we explored whether CGRP also increased in VVS patients.

Therefore, a comparison of the value of plasma CGRP among healthy children and children with VVS was conducted and the potential of this peptide as a predictive biomarker of the therapeutic effect of midodrine upon VVS was explored.

## Materials and methods

### Subjects

The sample size was estimated based on the primary evaluation parameter, which was the sensitivity (81%) and the specificity (92%) of CGRP in the pre-experiment. With α = 0.05 and δ = 0.1, the sample size of the case and control group is 60 and 29 respectively. The formula applied is as follows:


$$n={\left(\frac{z_{1-\text{α/2}}\;*\;\sqrt{p\;*\;\left(1-p\right)}}{\delta }\right)}^{2}$$

**FIGURE 1:**
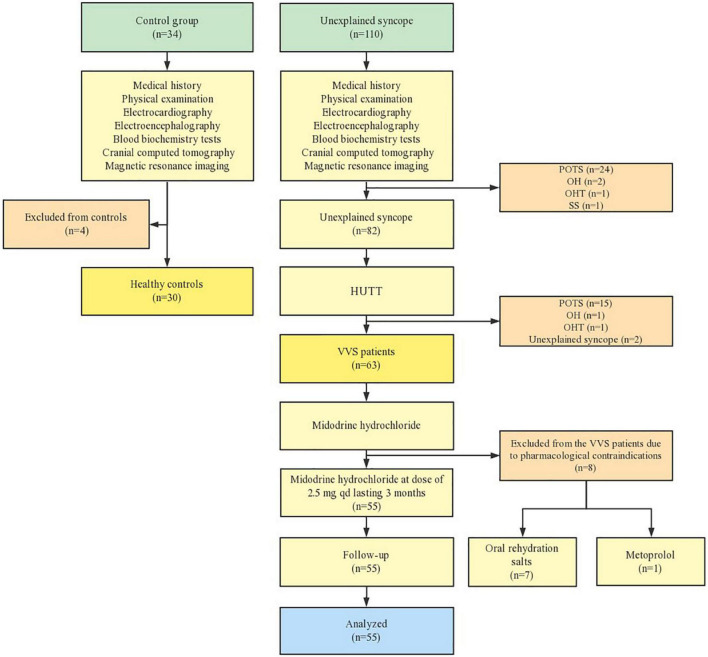
Flow chart of participants selection. POTS, postural tachycardia syndrome; VVS, vasovagal syncope; OH, orthostatic hypotension; OHT, orthostatic hypertension; SS, situational syncope; HUTT, head-up tile test.

Figure 1 summarized the sample loss during the research. Among the 93 children enrolled, 63 subjects were diagnosed with VVS and the remaining 30 were healthy. All subjects were enrolled during their visits of either the outpatient clinic or inpatient pediatric departments from July 2019 to June 2021 in Shaanxi Provincial People’s Hospital, China. In addition to a detailed medical history, physical inspection, and lab results such as electrocardiography, electroencephalography, blood biochemistry tests, as well as cranial computed tomography or magnetic resonance imaging, were applied to rule out psychological, neurological, metabolic, cardiovascular reasons, migraine disease and patients with other clinical conditions. Normal results including their medical history, physical examination, electrocardiography, electroencephalogram, and head-up tilt test (HUTT) were found among the 30 healthy children. The control group were matched as far as possible in terms of age, gender, height, weight, and residence location to minimize the confounding factor. The aim of this study was explained and informed consent was gained with the permission of subjects enrolled. Also, guardians of all subjects agreed and gave written informed consent. The ethics committees of Xi’an Jiaotong University, school of medicine approved of the present study (2018–84).

### Diagnostic criteria for vasovagal syncope

VVS in children was diagnosed according to the following criteria: (1) The occurrence of syncopal attack in older pediatric subjects; (2) Predispositions include persistent standing, quick change of physical position, heat, suffocating environment, or signs of nervousness; (3) at least one positive results in HUTT; and (4) ruling out complications that could induce loss of consciousness (Chinese Journal of Pediatrics et al., 2016).

### Head-up tilt test

VVS was diagnosed using the HUTT, which was conducted in a silent, dimly lit, warm environment. Prior to the HUTT, all children were fasted for over 4 h and were instructed to abstain from medications with effect on the function of autonomic system. All subjects were instructed to lay down on a tilt table (HUT-821; Beijing Juchi, China), and cardiovascular parameters were tracked with a Dash 2000 Multi-Lead Physiological Monitor (General Electric, New York, NY, USA). Starting with a stable heart rate, the subject would be instructed to lay on a table tilted at a 60° angle, and the subject’s heart rhythm and blood pressure would be recorded till the onset of a positive response or the end of the test which would last 45 min. A positive response in the HUTT was defined as follows: (1) after tilting, significant hypotension lasting for over 3 min could be detected (i.e., systolic blood pressure ≤ 80 mmHg, diastolic blood pressure ≤ 50 mmHg, or ≥ 25% reduction of mean blood pressure); (2) bradycardia (i.e., heart rate < 75 bpm in subjects aged 4–6 year, < 65 bpm in those aged 6–8 year, and < 60 bpm in those aged > 8 year); (3) second-degree or more severe atrioventricular block or asystole lasting over 3 s; and (4) cardiac arrest (Wang et al., 2018). HUTT was performed by a cardiologist who was blinded to the clinical history.

### Measurement of plasma calcitonin gene-related peptide content

Subjects were instructed to stay in the supine position, and venous blood specimen were collected from elbow vein for tests between 8 and 11 a.m. All blood samples were immediately centrifuged for 10 min at the speed of 2,000 rpm, blood plasma was collected and reserved at –80°C. ELISA assay kits purchased from USCN Life Science Inc. were used for the detection of the concentration of CGRP in plasma, and the assay was conducted strictly following the instructions provided by the manufacturer. A spectrophotometer (BioRad) was applied for detecting the level of absorption, with a limit detection level of CGRP at 4.3 pg/mL. The testers were blinded to the all the specimen to be tested.

### Treatment and follow-up

All subjects with confirmed diagnosis of VVS were treated with midodrine hydrochloride at the dose of 2.5 mg qd lasting 3 months. All subjects and their guardians agreed to notify the study staff any changes relative to clinical symptoms or medication administration. The data of all VVS subjects were analyzed based on the scorings of clinical symptoms before treatment and at the follow-up (3 months), when the HUTT was repeated. Among the 63 VVS patients, eight patients were not treated by midodrine hydrochloride due to pharmacological contraindications (allergy, bradycardia, hypertension, or insomnia etc.). Among these patients, one was administered with metoprolol [0.5 mg/(kg⋅d), bid], while the remaining seven received oral rehydration salts (NaCl 1.75 g, NaHCO_3_ 1.25 g, KCl 0.75 g, glucose 10 g; 500 ml qd) (Table 1). All eight patients were excluded from the follow-up.

**TABLE 1:** Treatment of patients with VVS.

Diagnosis	No. of cases	Medication	Length of the treatment
VVS	55	Midodrine hydrochloride. (2.5 mg, qd)	3 months
VVS	1	Midodrine hydrochloride. (2.5 mg, qd)	Midodrine hydrochloride: 3 days
		Metoprolol [0.5 mg/(kg⋅d), bid]	Metoprolol: 2 weeks
VVS	7	Midodrine hydrochloride. (2.5 mg, qd)	Midodrine hydrochloride: 3 or 4 days
		Oral rehydration salts (NaCl 1.75 g, NaHCO_3_ 1.25 g, KCl 0.75 g, glucose 10 g; 500 ml qd)	Oral rehydration salts: 4 weeks

VVS, vasovagal syncope.

### Criterion for the analysis of therapeutic efficacy

The therapeutic effect of midodrine hydrochloride was evaluated using a scoring system with regards to the 10 canonical symptoms of orthostatic intolerance associated with VVS: clouding of consciousness, syncope, headaches, trembling hands, cardiac palpitations, perspiration, chaotic state and the blurring of vision. The scorings of clinical symptoms manifested were recorded as below: without VVS symptoms were scored zero point; one onset of one symptom within 30 days was scored 1 point; 2–4 times onsets of one symptom within 30 days were scored 2 points; 2–7 times onsets of one symptom within 7 days were scored 3 points; and onsets of one symptom for more than once a day were scored 4 points. The total score for each participant was based on the sum of symptom scores. Symptom scoring was applied to all subjects once enrolled in the study and also after 3 months of treatment. Responders and non-responders were defined as those participants with a decrease in the total symptom score 2 points or < 2 points after treatment, respectively (Wang et al., 2018).

### Statistical analysis

All data analysis in this study were calculated by SPSS (version 19.0, IBM Corp, Armonk, Chicago, USA). Categorical data were described as the number of cases, and continuous data were described as the mean ± SD. Comparisons of categorical data were analyzed using χ^2^-test. Comparisons of continuous data prior to and post medication of individuals were analyzed by a paired *t*-test. The value of CGRP concentrations in predicting the efficacy of midodrine hydrochloride was analyzed by the receiver-operating characteristic (ROC) curve. An area under the ROC curve (AUC) of 0.5–0.7 indicates low predictive value; AUC of 0.7–0.9 indicates moderate predictive value; and AUC > 0.9 indicates high predictive value. The cut-off value was defined as the maximal Youden index, which is defined as the sensitivity plus specificity minus 1. *P* < 0.05 was defined as statistical significance.

## Results

### Clinical features of vasovagal syncope subjects and control subjects

In total, 63 VVS patients (24 male), and 30 healthy controls (15 male) were recruited in this study. The mean age of the children diagnosed with VVS and healthy controls was 13.11 and 12.60 year, respectively. The clinical features and hemodynamic data of VVS subjects and healthy subjects are shown in Table 2. Before treatment, no difference of gender, age, height, weight, supine HR or supine blood pressure between the two groups was found with statistical significance (*P* > 0.05). However, CGRP levels were more potently increased in patients diagnosed with VVS (68.700 ± 6.460) compared with those in the control group (43.400 ± 5.810; *t* = 18.207, *P* < 0.001).

**TABLE 2:** Clinical features and hemodynamic data of VVS subjects and control subjects.

	VVS	Control	*t*/χ ^2^	*P*
Gender (male/female)	24/39	15/15	1.183	0.277
Age (years)	13.110 ± 1.850	12.600 ± 1.480	1.430	0.156
Height (cm)	148.970 ± 12.530	151.130 ± 11.910	0.791	0.431
Weight (kg)	42.080 ± 8.240	38.950 ± 9.510	1.632	0.106
Supine HR (beats/min)	77 ± 7	76 ± 6	0.496	0.621
Supine SBP (mmHg)	102 ± 7	101 ± 7	0.671	0.504
Supine DBP (mmHg)	63 ± 7	61 ± 6	1.390	0.168
CGRP (pg/ml)	68.700 ± 6.460	43.400 ± 5.810	18.207	<0.001

Data were expressed as means ± SEM. CGRP, calcitonin gene-related peptide; DBP, diastolic blood pressure; HR, heart rate; SBP, systolic blood pressure; VVS, vasovagal syncope.

### Relationship between plasma calcitonin gene-related peptide concentrations and the severity of clinical symptoms

**FIGURE 2:**
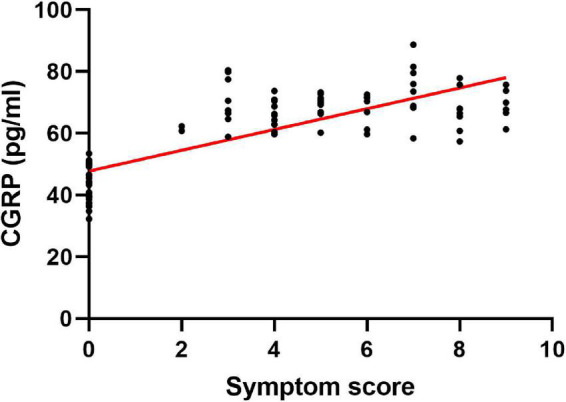
Relationship between the plasma level of CGRP and the severity of clinical symptoms. Symptom scores correlate positively with plasma CGRP concentrations. CGRP, calcitonin gene-related peptide.

In our analysis of the value of plasma CGRP concentrations as a biomarker of VVS severity, we found that symptom scores correlated with CGRP concentrations positively (*r* = 0.779, *P* < 0.001) (Figure 2).

### The symptom scoring and hemodynamic parameters of children with vasovagal syncope

**FIGURE 3:**
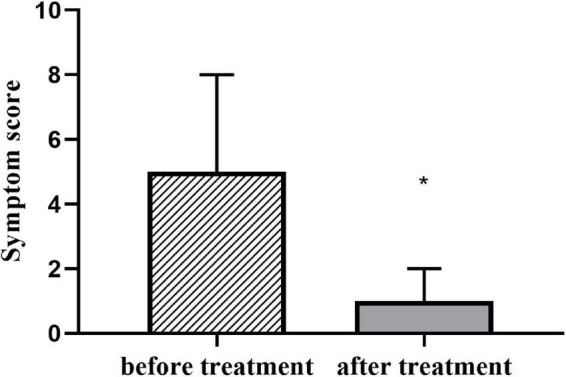
Symptom score in patients with VVS before and after treatment with midodrine hydrochloride [4(0, 6.5) vs. 1(1, 2); z = –6.481; *P < 0.001]. VVS, vasovagal syncope.

A total of 55 subjects diagnosed with VVS were treated with midodrine hydrochloride (2.5 mg/day) once a day for 3 months, and with the scoring of symptoms re-evaluated, the HUTT was repeated. The symptom scores of patients were significantly decreased with treatment (Table 3 and Figure 3).

**TABLE 3:** Analysis of symptom scores and hemodynamic parameters of VVS patients treated by midodrine hydrochloride.

	Before treatment	After treatment	*t/z*	*P*
Symptom score (Median, Q1,Q3)	4 (0, 6.5)	1 (1, 2)	–6.481	<0.001
Supine HR (beats/min)	77 ± 8	80 ± 7	2.900	0.005
Supine SBP (mmHg)	101 ± 7	101 ± 7	0.442	0.661
Supine DBP (mmHg)	63 ± 7	61 ± 6	1.699	0.095
HR at positive response	93 ± 31	81 ± 6	2.731	0.009
Occurrence in HUTT (beats/min)				
SBP at positive response	99 ± 13	107 ± 9	4.820	<0.001
Occurrence in HUTT (mmHg)				
DBP at positive response	64 ± 10	68 ± 6	3.357	0.001
Occurrence in HUTT (mmHg)				

Data were expressed as means ± SEM, or median (interquartile ranges). DBP, diastolic blood pressure; HR, heart rate; HUTT, head-up tilt test; SBP, systolic blood pressure.

### Children with vasovagal syncope treated with midodrine hydrochloride

Of the 55 patients with VVS, 43 were classified as responders and 12 were classified as non-responders. There were no differences in HUTT results between the two groups, with the exception of heart rate and systolic blood pressure during a positive reaction. However, plasma CGRP levels were significantly higher in the responders compared with those in non-responders (70.080 ± 5.040) vs. (61.150 ± 3.090); *t* = 5.817; *P* < 0.001 (Table 4).

**TABLE 4:** Analysis of demographic data and clinical features in VVS subjects treated with midodrine hydrochloride.

	Responder	Non-responder	*t/*χ^2^/z	*P*
Gender (male/female)	18/25	3/9	1.130	0.288
Age (years)	13.140 ± 1.780	13.420 ± 1.880	0.471	0.640
Height (cm)	148.050 ± 12.080	150 ± 13.500	0.483	0.631
Weight (kg)	41.510 ± 7.430	42.670 ± 10.250	0.437	0.664
Symptom duration before	20.580 ± 7.360	18.080 ± 5.400	1.094	0.279
Treatment (months)				
Symptom scores before	6 (4, 7)	4.5 (3.25, 8)	–0.763	0.445
Treatment (Median, Q1,Q3)				
Symptom scores at	1 (0.25, 2)	2.5 (2, 6.75)	–3.441	0.001
Follow-up (Median, Q1, Q3)				
Treatment duration (months)	2.880 ± 0.680	3.060 ± 0.670	1.704	0.095
Supine HR (beats/min)	78 ± 7	75 ± 9	1.088	0.282
Supine SBP (mmHg)	102 ± 7	99 ± 8	1.385	0.172
Supine DBP (mmHg)	63 ± 7	62 ± 8	0.416	0.679
HR at positive response	98 ± 29	74 ± 32	2.522	0.015
Occurrence in HUTT (beats/min)				
SBP at positive response	101 ± 11	92 ± 18	2.137	0.037
Occurrence in HUTT (mmHg)				
DBP at positive response	65 ± 8	59 ± 15	1.824	0.074
Occurrence in HUTT (mmHg)				
CGRP (pg/ml)	70.080 ± 5.040	61.150 ± 3.090	5.817	<0.001

Data were expressed as means ± SEM, or median (interquartile ranges). CGRP, calcitonin gene-related peptide; DBP, diastolic blood pressure; HR, heart rate; HUTT, head-up tilt test; SBP, systolic blood pressure; VVS, vasovagal syncope.

### Value of plasma calcitonin gene-related peptide

**FIGURE 4:**
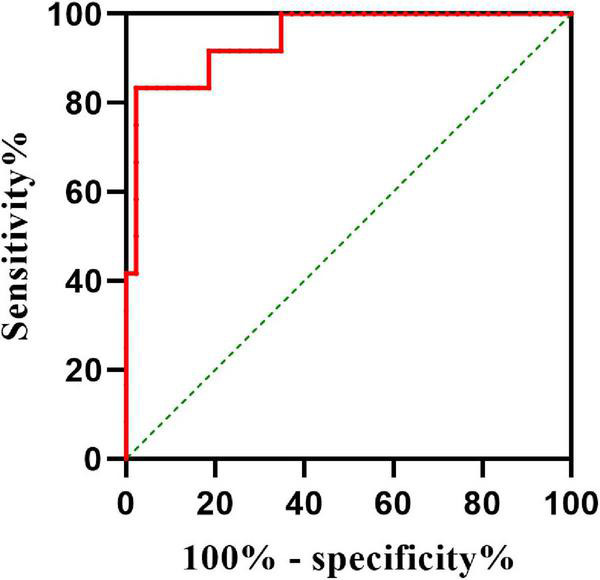
Value of plasma CGRP in predicting the therapeutic efficacy of midodrine hydrochloride in children with VVS. The y-axis represents the sensitivity of the CGRP for predicting the effectiveness of midodrine hydrochloride therapy. The x-axis represents the false positive rate (1-specificity) of the prediction. The intermittent green line indicates the point at which the sensitivity is equal to false positive rate (i.e., no predictive value). The red line represents the ROC curve (AUC 94.6%, 95% CI 87.9–99.7%) indicating that pre-treatment plasma CGRP levels have a high predictive value in assessing the efficacy of midodrine hydrochloride in patients with VVS. CGRP, calcitonin gene-related peptide; VVS, vasovagal syncope; ROC, receiver-operating characteristic curve; AUC, area under curve.

To determine the value of plasma CGRP as a predictive biomarker of the efficacy of midodrine hydrochloride, we calculated the AUC as 0.946 (95% CI: 0.879–0.997, *P* < 0.001). Plasma CGRP concentration predicted treatment efficacy with high sensitivity (97.7%) and specificity (83.3%) at a cut-off value of 62.56 pg/ml (Figure 4).

## Discussion

In our study, we found that the level of plasma CGRP in pediatric subjects diagnosed with VVS was markedly elevated compared to that of the healthy subjects. Furthermore, plasma CGRP levels correlated positively with the severity of clinical symptoms in VVS patients, indicating that plasma CGRP levels before treatment can be used to evaluate the efficacy of treatment with midodrine hydrochloride.

VVS is the most common reason for attendance at the emergency department in school-age children, with a higher incidence in female than male. It is often characterized by sudden syncope during prolonged periods of standing or standing suddenly, which can induce a brief episode of dizziness, confusion, pallor, loss of vision or hearing, nausea, vomiting or sweating. Some severe cases will lose consciousness briefly (10–20 s) and collapse. Although the prognosis of most patients affected by VVS is good, recurrent episodes of syncope affect their quality of life, with some patients dropping out of school and unable to attend social activities. VVS often leads to injury due to the loss of consciousness, and can be at risk of sudden death (Grubb, 2005b). Therefore, an effective treatment is urgently required to improve symptoms in children with VVS.

The major problems in management strategies for VVS may be that the therapeutic options do not specifically target the mechanisms in patients with different primary pathogeneses, and there are no indicators for the various mechanisms to facilitate selection (Liao and Du, 2020). It is necessary to find predictors of the therapeutic effect in VVS patients that indicate the dominant pathophysiological mechanism in each patient, to identify individuals who will respond to the corresponding therapy and consequently improve the quality of life for VVS patients who are affected by recurrent syncope.

The possible mechanisms of VVS include genetic factors, an abnormal Bezold–Jarisch reflex, vasomotor dysfunction, central hypovolemia and autonomic nervous dysfunction. Hence, children with VVS have received salt, water, betablockers, or midodrine as treatments. However, the efficacy of the drugs varies. Midodrine hydrochloride is a selective alpha-adrenergic receptor agonist which could increase the resistance in peripheral vasculatures and reduce venous pooling. Due to the crucial effect of vasodilatation in the pathogenetic development of VVS, such peripherally-acting agents were considered to attenuate susceptibility to VVS. In addition, midodrine does not penetrate the blood–brain barrier, resulting in no central nervous system side effects. Midodrine is an approved agent for orthostatic hypotension management in the United States (Low et al., 1997), the beneficial effects of midodrine have been confirmed in a series of studies on adult VVS (Samniah et al., 2001; Kaufmann et al., 2002). However, the efficacy of midodrine hydro-chloride in children and adolescents is variable (Mitro et al., 1999; Kaufmann et al., 2002; Qingyou et al., 2006; Romme et al., 2011; Bagrul et al., 2021). Great improvements were achieved in terms of individualized therapies and before the application of any treatment, biological markers or predictors could provide useful information for doctors to choose a specific treatment regimen. The changes of the heart rate during the HUTT, LVFS (left ventricular fractional shortening) and LVEF (left ventricular ejection fraction), Baroreflex sensitivity (BRS) as well as 24 h urine norepinephrine were all effective indicators to predict β-Blocker efficacy in pediatric VVS, however, during the past years, FMD is the only effective predictor for predicting VVS with midodrine so far. In 2012, Zhang et al. explored the application of FMD of brachial artery for prediction of the therapeutic effect of midodrine hydrochloride among pediatric patients with VVS (Zhang et al., 2012a). FMD of the brachial artery was identified to be strongly elevated in VVS patients showing good responses to treatment compared with those who responded poorly. ROC curve analysis of the predictive value of FMD of the brachial artery revealed that the AUC was 0.895. Using a cut-off of 8.85%, FMD was shown to predict the efficacy of midodrine hydrochloride administration in VVS subjects with high sensitivity (90%) and specificity (80%). This index provides great help for clinical guidance of individualized treatment for VVS patients. However, we wanted to explore whether there were better predictors than FMD. Besides, only 24 children were enrolled in Zhang’s study, and the response to treatment was evaluated in-person after only 1 month of treatment, with the telephone follow-up conducted after 3 months. We demonstrated that plasma CGRP concentration predicted treatment efficacy with high sensitivity (97.7 %) and specificity (83.3%) at the cut-off value of 62.56 pg/ml. All the patients in our study were treated for 3 months, when the symptom scores were re-evaluated and the HUTT repeated. Thus, our findings demonstrate the greater value of plasma CGRP levels for predicting midodrine hydrochloride treatment efficacy in patients with VVS.

CGRP was first discovered in 1982 and was soon identified as an important vasodilator and neurotransmitter, especially in the nervous systems. Produced in sensory nerve terminals, CGRP primarily innervates arterial and venous vasculatures following its release via several mechanisms of depolarization (Sohn et al., 2020). CGRP-positive nerves are mainly localized in unmyelinated C fibers and thinly myelinated A δ-fibers in the adventitia of arteries and veins. Chemically, CGRP is released after activating the vanilloid TRPV1 receptor. Electrically, CGRP could be released by stimulated nerve and induces vasodilation in the nanomolar range among various species and arteries. Intravenous infusion of CGRP could result in hypotension (Struthers et al., 1986) and increased blood supply to the skin and brain (Jäger et al., 1990). Qingyou et al. (2005). reported that increase FMD and vasodilation in children with VVS. Based on our observation that plasma CGRP levels were significantly increased in children with VVS, we hypothesize that vasodilation in patients with VVS is closely related to increased plasma CGRP levels.

In addition to vasomotor dysfunction, an abnormal Bezold–Jarisch reflex is also considered to be an important potential mechanism of VVS. This reflex involves a downward blood flow of approximately 300–800 mL to the blood vessels in the abdominal and lower extremities, promoting the sympathetic outflow. If adequate vascular tone is not maintained, the blood volume returned to the heart continues to decrease, the sympathetic tension increases further, and the C fibers in the ventricular wall are activated. The impulses transmitted by the C fiber increase vagus nerve activity in the brain stem, inhibit sympathetic nerve activity, and cause peripheral vasodilation. Consequently, blood pressure drops and bradycardia may occur, resulting in reduced blood supply in the cerebral system and fainting. We hypothesize that VVS patients experience a recurrent syncope due to Bezold–Jarisch reflex, which may be related with the release of CGRP from C fibers (Mosqueda-Garcia et al., 1997). Further studies are required to confirm the direct relationship between C fiber in the posterior inferior myocardial wall and plasma CGRP levels in patients with VVS.

While the hemodynamic parameters of the responder and non-responder groups differed only slightly before treatment, the plasma CGRP levels of the two groups were significantly different. Nonetheless, we need more investigations in future to fully elucidate the molecular mechanisms involving that with higher CGRP values, responses to midodrine hydrochloride were better.

Dysfunctional autonomic nervous system, dysfunctional vasomotor, aberrant Bezold–Jarisch reflex, genetic factors, in addition to abnormal cerebrovascular autoregulation as well as neurohumoral factors (including catecholamines, 5-hydroxytryptamine, nitric oxide, and adenosine) are all considered potential mechanisms for VVS (Kaufmann et al., 1991; Halliwill et al., 1996; Liu et al., 2000; Mosqueda-Garcia et al., 2000; Savelieva and Camm, 2008); thus, mechanistic differences may account for the variation in treatment responses of children with VVS in our research.

Previous studies have focused on vasodilation induced by gas signaling molecules in VVS. Previous studies have indicated that plasma nitric oxide and increased H_2_S production by erythrocytes may be associated with the pathogenetic development of VVS in pediatric patients (Yang et al., 2015). Ours is the first study focused on the plasma CGRP levels in patients with VVS and its value as a predictor of the therapeutic efficacy of midodrine hydrochloride, which can be used to determine individualized treatment. Since CGRP is metabolized rapidly, further clarification of the dynamic changes in CGRP levels in patients with VVS is required to fully understand its value as a predictive biomarker. In addition, the release of CGRP could be triggered by various stimuli; therefore, it is important to determine the type of stimulation that causes increased plasma levels of CGRP in VVS patients.

Notably, there are a few limitations in the present study. Since this study is single-centered with a relatively small number of patients and a short follow-up, in future, we should consider large sample size, multi-center studies with long follow-up. Similar to other peptides, CGRP is cleared via glomerular filtration with a short plasma half-life of 7 min. Therefore, although blood samples were collected soon after the HUTT test, these samples may not represent the peak plasma CGRP concentrations. At last, the FMD should be applied to the VVS patients during the follow-up period so that to prove that the treatment effects of midodrine hydrochloride in VVS patients may be are mediated by its ability to facilitate vasoconstriction, which counteracts the vasodilation induced by the elevated plasma CGRP levels.

## Conclusion

The present study reveals that the symptoms of VVS patients could be improved by midodrine hydrochloride which is expected and consistent with past researches. With a high sensitivity and specificity, plasma level of CGRP may serve as a useful index predicting the therapeutic response of midodrine hydrochloride in VVS patients. Further studies are apparently needed to confirm our findings on the increment of CGRP in VVS patients and the molecular mechanisms involving that with higher CGRP values, responses to midodrine hydrochloride were better as well.

## Data availability statement

The raw data supporting the conclusions of this article will be made available by the authors, without undue reservation. For more information, contact the corresponding author at linjing0127@xjtu.edu.cn.

## Ethics statement

The studies involving human participants were reviewed and approved by Xi’an Jiaotong University, School of Medicine. Written informed consent to participate in this study was provided by the participants’ legal guardian/next of kin.

## Author contributions

FJ and JL: conceptualization and project administration. HZ and LL: data curation and software. XM and LL: formal analysis. HZ and JL: funding acquisition. XM: investigation, visualization, and writing–original draft. XM and JL: methodology. JL: resources and supervision. XM and HZ: validation. HZ, FJ, and JL: writing–review and editing. All authors contributed to the article and approved the submitted version.
